# Topology-Driven Compression and Energy Absorption of PLA-Lattice-Reinforced Mortar

**DOI:** 10.3390/ma19183932

**Published:** 2026-09-16

**Authors:** Miguel Loyola, César Garrido, Marian Valenzuela, Víctor Tuninetti, Rodrigo Valle

**Affiliations:** 1 Construction Multidisciplinary Research Group, Facultad de Arquitectura, Construcción y Medio Ambiente, Universidad Autónoma de Chile, Talca 3460000, Chile; miguel.loyola@cloud.uautonoma.cl; 2 Department of Mechanical Engineering, Universidad del Bío-Bío, Concepción 4081112, Chile; cgarrido@ubiobio.cl; 3 Doctoral Program in Sciences of Natural Resources, Universidad de La Frontera, Casilla 54-D, Temuco 4780000, Chile; m.valenzuela16@ufromail.cl; 4 Sustainable Building Design Lab, Department UEE, Faculty of Applied Sciences, University of Liège, 4000 Liege, Belgium; 5 Department of Mechanical Engineering, Universidad de La Frontera, Temuco 4811230, Chile

**Keywords:** architected cementitious composite, auxetic structures, mortar reinforcement, energy absorption, matrix confinement

## Abstract

The design of cellular architectures based on topology offers a promising strategy for tailoring the mechanical performance of cementitious composites without altering the matrix composition. However, the influence of the topological transition from re-entrant auxetic structures to conventional honeycomb structures on the compressive response of mortar is not yet fully understood. In this study, a family of polylactic acid (PLA) cellular architectures was systematically designed using fused deposition modeling (FDM), varying the strut angle from $-70^{\circ }$ to $+70^{\circ }$ and thereby generating a transition from auxetic to honeycomb topologies, with the cubic configuration serving as the intermediate topology. Quasi-static compression tests were conducted on standalone lattices, lattice-reinforced mortar composites, and unreinforced mortar specimens. The response was characterized in terms of apparent compressive modulus, apparent yield stress, energy absorption density, and specific energy absorption. Embedding the PLA lattices in mortar changed the macroscopic post-yield response, with the reinforced specimens sustaining deformation over a larger strain interval than the corresponding isolated lattices. This behavior is consistent with a constraint effect imposed by the surrounding matrix; however, the post-test PLA–mortar interface condition, possible debonding or delamination, and internal crack distribution were not directly characterized. Accordingly, the compressive response of these structured composites is governed by the combined effects of cellular topology and matrix–lattice interaction, while the specific microscale mechanisms underlying this interaction require direct experimental validation. These findings establish topological transition as a design strategy for developing cementitious composites with tailored quasi-static mechanical performance.

## 1. Introduction

Cementitious materials are widely used in structural and nonstructural applications because of their availability, manufacturing versatility, and high compressive load-carrying capacity [1,2]. Their mechanical response is nevertheless governed by the nucleation, interaction, and localization of distributed microcracks, resulting in limited tensile deformability and an unstable transition toward macroscopic fracture [3]. This almost brittle behavior limits its applicability in situations that require controlled deformation, progressive load redistribution, impact resistance, or efficient dissipation of mechanical energy [4,5].

Conventional strategies for modifying the mechanical response of cementitious materials generally rely on changes in matrix composition, including supplementary cementitious materials, chemical admixtures, modified aggregates, dispersed fibers, or continuous reinforcement [6,7]. While these approaches have successfully improved specific mechanical properties, they require modifications to the cementitious material formulation [8]. Furthermore, their performance can be influenced by factors such as the dispersion and orientation of the reinforcement, interfacial adhesion, and manufacturing quality [9,10]. An alternative strategy involves preserving the original composition of the matrix while tailoring the mechanical response through the precise design of the material’s internal architecture [11,12].

Recent advances in additive manufacturing have enabled the production of cellular materials with defined architectures, whose overall mechanical behavior is governed primarily by geometry rather than by the properties of their components [13]. Unlike conventional solid materials, cellular architectures derive their apparent stiffness [14], instability modes [15], and energy absorption [16] characteristics from parameters such as topology, strut orientation, nodal connectivity, and relative density [17,18,19]. This design freedom has established structured materials as an attractive tool for developing new structures with programmable mechanical behavior [20]. Depending on the selected topology, the load-transfer mechanism may be dominated by axial tension, bending, rotation, or progressive buckling, resulting in substantially different combinations of stiffness, strength, and energy dissipation [21,22,23]. Recent studies have shown that cellular structures produced using additive manufacturing can also serve as effective internal reinforcements in composite materials [24,25,26]. Their incorporation has been explored in polymer matrices, such as thermosetting resins, where the integrated cellular network improves stiffness, damage tolerance, and failure mechanisms [27,28]. Similarly, cellular architectures have been integrated into polyurethane foams and polyurea-based composites to improve impact resistance and energy absorption through the controlled deformation of the structured reinforcement [29,30]. More recently, this design concept has been extended to cementitious materials, where embedded polymeric cellular architectures have demonstrated their potential to modify compressive behavior while maintaining the mortar’s original composition [31,32,33]. Collectively, these studies demonstrate that cellular architectures can actively contribute to the overall mechanical response of composite systems, highlighting topology as an additional design variable beyond the intrinsic properties of the constituent materials.

Despite these advances, existing studies have focused primarily on evaluating individual cellular configurations. Consequently, relatively little attention has been paid to understanding how a systematic topological transition influences the mechanical response of cementitious composites. In particular, the effect of progressively varying the angle of the struts—from re-entrant auxetic architectures to conventional honeycomb architectures—has not been experimentally investigated under identical material and manufacturing conditions. Since the strut angle directly governs load paths, deformation mechanisms, and structural anisotropy, understanding its influence is essential for establishing topology-based design guidelines for structured cementitious composites. Re-entrant lattices are commonly associated with auxetic kinematics because their internal members can rotate or bend in a manner that produces a negative effective Poisson’s ratio [34,35]. Such configurations have been investigated because of their indentation resistance, shear response, deformation control, and energy-absorption potential [36,37]. In contrast, conventional honeycomb structures generally have a positive Poisson’s ratio and exhibit comparatively more direct compressive load paths as the struts progressively align with the load direction [38,39]. Although auxetic and honeycomb lattice structures are usually studied as distinct structural concepts, they can also be interpreted as two limiting configurations within a continuous topological space [40,41]. The systematic variation of the strut angle provides a simple yet powerful geometric design parameter that allows for a continuous transition from re-entrant auxetic architectures to conventional honeycomb architectures, while preserving the same underlying cellular concept [42,43]. This topological transition progressively modifies the internal load paths, deformation mechanisms, and structural anisotropy without altering either the constituent material or the manufacturing process [44,45]. Consequently, it provides a rational framework for topology-based mechanical design, in which the influence of architecture can be isolated from changes in material composition. Despite this potential, relatively few studies have investigated the topological transition as a design strategy for cementitious composites reinforced with embedded cellular structures.

To address this knowledge gap, this study systematically investigates the influence of a topological transition from re-entrant auxetic architectures to conventional honeycomb structures on the quasi-static compressive behavior of mortar composites. A family of polylactic acid (PLA) cellular architectures was generated using fused deposition modeling (FDM), by varying only the strut angle, which produced a continuous transition from auxetic ($-70^{\circ }$ and $-50^{\circ }$) through a cubic configuration ($90^{\circ }$) to conventional honeycomb geometries ($+50^{\circ }$ and $+70^{\circ }$). This topology-based design framework allows for isolating the effect of the cellular architecture while keeping the constituent materials and fabrication conditions identical. Independent lattices, lattice-reinforced mortar composites, and unreinforced mortar specimens were experimentally evaluated to determine how topology influences the apparent compressive modulus, apparent yield stress, energy absorption density, and specific energy absorption, while also providing insight into the macroscopic mechanical interaction between the embedded cellular architectures and the surrounding cementitious matrix.

This article is organized as follows. Section 2 describes the topological design of the cellular architectures, the fabrication of the PLA lattices and the mortar composite specimens, as well as the experimental procedures adopted for the quasi-static compression tests. Section 3 presents and analyzes the experimental results, examining how the transition from re-entrant auxetic architectures to conventional honeycomb architectures influences the mechanical response of both the standalone lattices and the mortar-reinforced composites. Finally, Section 4 summarizes the main findings of the study and highlights their implications for the topological design of structured cementitious composites.

## 2. Materials and Methods

**Figure 1:**
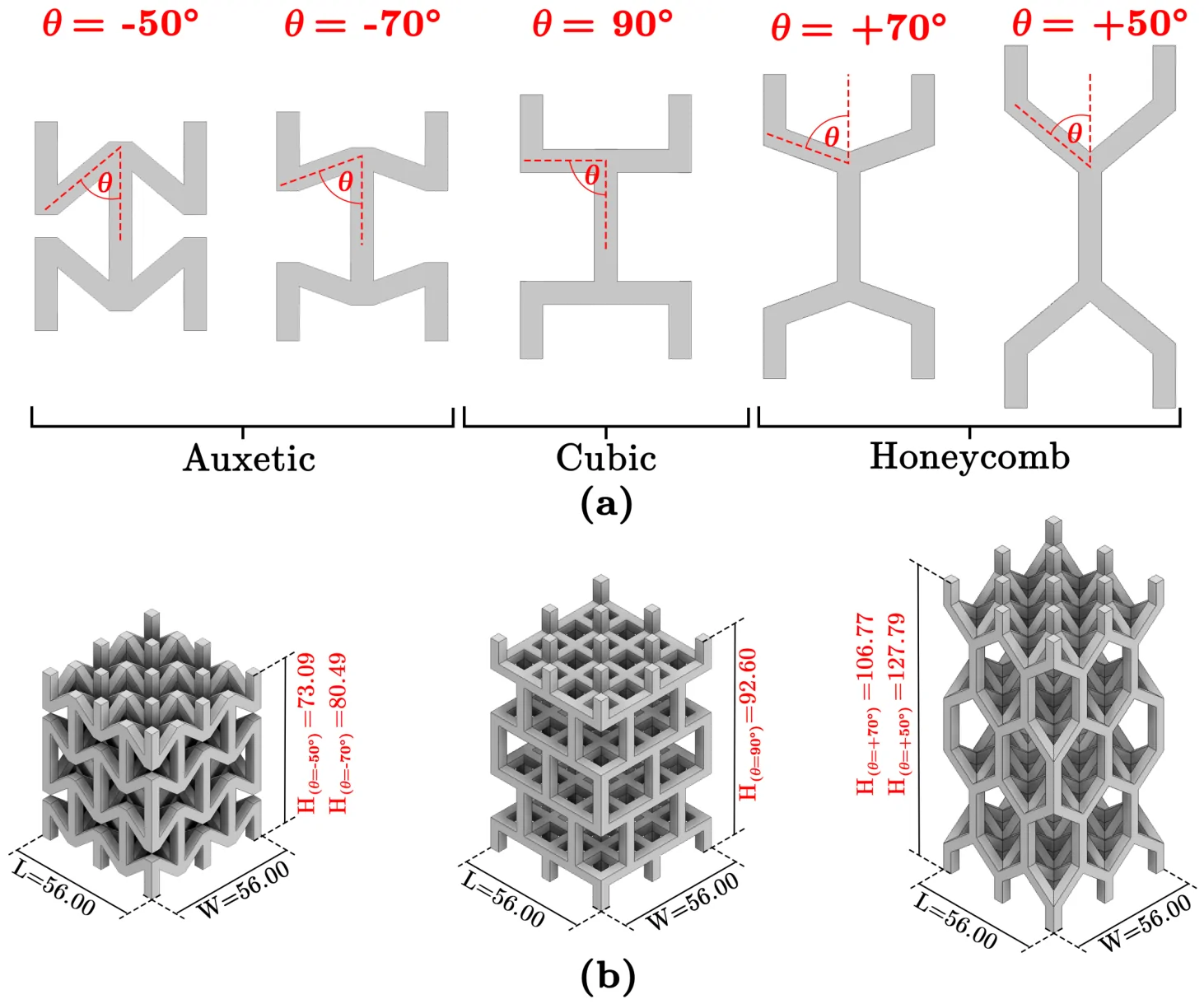
Geometric parameterization of the cellular reinforcements. (**a**) Unit-cell architectures obtained by systematically varying the strut angle (θ) from auxetic ($\theta =-70^{\circ }$ and $-50^{\circ }$), through a conventional cubic configuration ($\theta =90^{\circ }$), to honeycomb geometries ($\theta =+70^{\circ }$ and $+50^{\circ }$). (**b**) Global dimensions of the investigated cellular structures. All dimensions are expressed in millimeters (mm).

This study investigates the influence of cellular structure on the compressive behavior of cementitious composites reinforced with printed three-dimensional (3D) polymeric structures. The proposed reinforcement strategy consists of incorporating periodic cellular structures into a mortar matrix to modify load transfer, post-yield deformation, and energy dissipation without changing the mortar formulation. To isolate the effect of cellular topology, a parametric design approach was adopted in which the strut angle (θ), which defines the geometry of the unit cell, was systematically varied. Five cellular configurations were considered, comprising two auxetic architectures ($\theta =-70^{\circ }$ and $-50^{\circ }$), one conventional cubic architecture ($\theta =90^{\circ }$), and two honeycomb architectures ($\theta =+70^{\circ }$ and $+50^{\circ }$), as shown in Figure 1a. This geometric parameterization allowed for a direct evaluation of how the transition from re-entrant (Auxetic) topologies to conventional Honeycomb topologies influences the mechanical response of both the independent cellular structures and the corresponding mortar-cellular composites under uniaxial compression. Thus, the overall dimensions of the reinforcement such as length (*L*) and width (*W*) remained constant, while height (*H*) varied depending on the angle θ of the struts, as shown in Figure 1b.

The geometric parameters used in this study are illustrated in Figure 1. The total length (L= 56 mm) and width (W= 56 mm) of all cellular reinforcements were kept constant to ensure an identical contact area within the mortar specimens. Likewise, the dimensions of the unit cell, the strut thickness, the number of cells, and the casting material remained unchanged throughout the experimental program. Therefore, the angle of the struts (θ) was the primary geometric design variable used to generate the transition between the cellular architectures studied. However, variations in θ inherently produced coupled changes in the height of the lattice, the specimen volume, the bulk density, the mortar volume, and the volumetric fraction of the reinforcement. Consequently, these parameters cannot be considered entirely independent variables, and the mechanical differences observed among the configurations studied are interpreted as the result of the combined influence of the cellular geometry and its associated dimensional and density characteristics, rather than solely of the topology.

The angles of the selected struts were chosen based on previous research on structured cellular materials, which demonstrated that the mechanical response is strongly determined by the cellular topology and the deformation mechanism [34,46]. Recessed geometries with negative strut angles exhibit auxetic behavior, characterized by lateral expansion under compressive loading due to a deformation mechanism dominated by rotation [47]. This behavior promotes the progressive collapse of the cells, improves load redistribution, and increases energy absorption. Furthermore, previous studies have shown that auxetic lattices with $\theta =-70^{\circ }$ and relatively low strut length-to-thickness ratios exhibit high structural stiffness while maintaining stable progressive deformation [28], resulting in significantly improved energy absorption when embedded in cementitious matrices [31]. In contrast, positive strut angles generate conventional honeycomb-type architectures, whose mechanical response is primarily determined by the bending of the inclined struts and localized buckling [48]. Although these structures generally have lower initial stiffness than re-entrant trusses, they offer a stable crushing response and are widely recognized as lightweight and efficient cellular architectures due to their excellent stiffness-to-weight ratio and effective load distribution [49,50]. Consequently, honeycomb structures constitute a suitable reference topology for comparison with auxetic reinforcements. The conventional cubic architecture ($\theta =90^{\circ }$) was included as an intermediate reference configuration that bridges the re-entrant and honeycomb topologies.

This experimental design allowed for a comparative evaluation of the mechanical response associated with the geometric transition between the cellular architectures under investigation, while keeping the constituent material, the dimensions of the unit cell, the thickness of the struts, the number of cells, and the planar dimensions constant. However, since variations in the angle of the struts also produced coupled changes in the specimen height and density-related characteristics, the observed response reflects the combined influence of the cellular architecture and these associated variations. This approach allows for a direct evaluation of how the deformation mechanisms associated with auxetic, cubic, and honeycomb architectures affect load transfer, stiffness, post-yield response, and energy dissipation when cellular structures are integrated into the mortar matrix.

### 2.1. Fabrication of the Cellular Reinforcements

**Figure 2:**
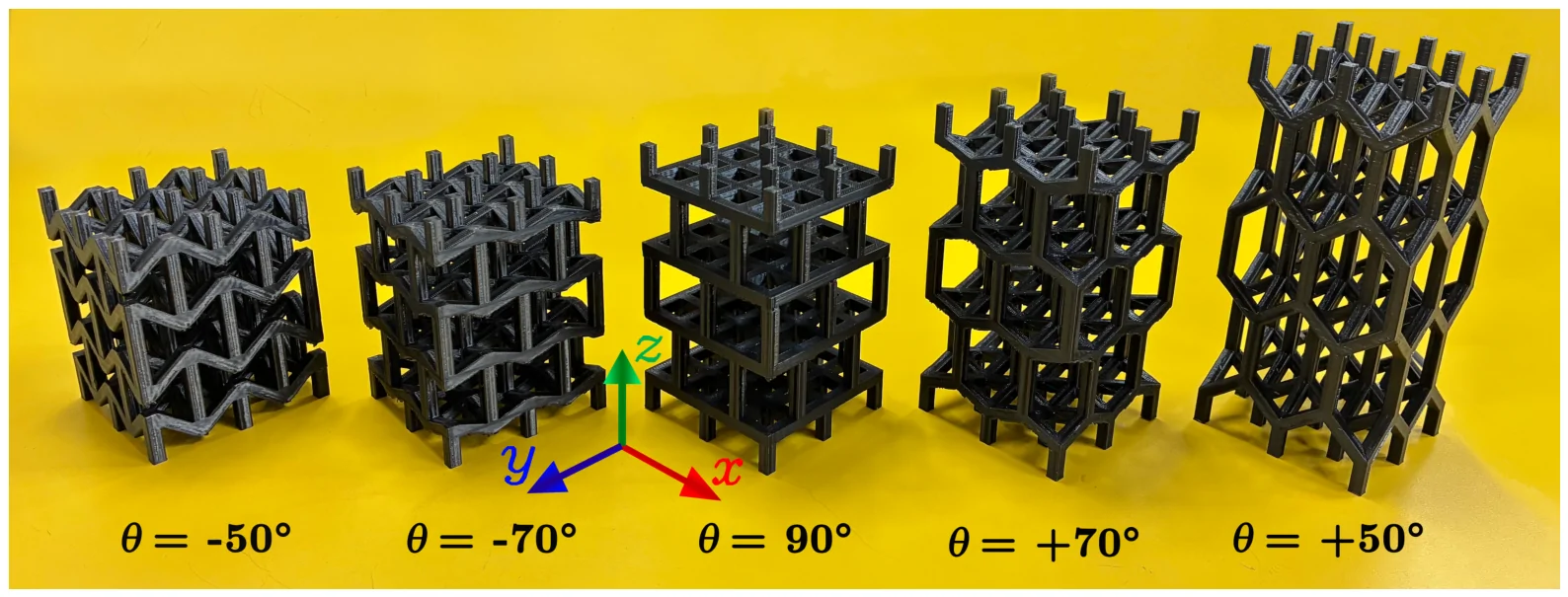
Representative FDM-printed PLA cellular reinforcements corresponding to the five investigated topologies. The specimens were manufactured under identical processing conditions, while the strut angle (θ) was systematically varied to obtain auxetic, cubic, and honeycomb architectures.

The cellular structures were fabricated using fused deposition modeling (FDM) with a Creality Ender 3 S1 Pro 3D printer (Shenzhen Creality 3D Technology Co., Ltd., Shenzhen, China) and commercial Creality polylactic acid (PLA) filament. To ensure manufacturing uniformity and minimize process-induced variability, all samples were fabricated under identical printing conditions. Printing parameters included a nozzle temperature of 210 °C, a build plate heated to 50 °C, a constant print speed of 70 mm/s, and a layer height of 0.20 mm. Furthermore, all cellular structures were printed directly onto the build plate with their longitudinal axis *z* aligned parallel to the xy plane, as illustrated in Figure 2, ensuring identical build orientation and interlayer deposition for each configuration. Consequently, the print orientation and the associated FDM-induced anisotropy were maintained as controlled and identical manufacturing conditions for all cellular configurations. Therefore, the potential effects associated with interlayer bonding were common to all the topologies investigated and were not considered independent variables in this comparative study.

Figure 2 shows the five fabricated topologies. The configurations investigated include two auxetic architectures with strut angles of $\theta =-70^{\circ }$ and $\theta =-50^{\circ }$, a conventional cubic architecture ($\theta =90^{\circ }$), and two honeycomb architectures with $\theta =+70^{\circ }$ and $\theta =+50^{\circ }$. The fabricated samples accurately reproduced the designed cellular geometries, maintaining the geometric characteristics defined in the CAD models. However, to improve the accuracy of the results, they were measured with a digital caliper, using the actual fabrication dimensions to calculate the stresses.

For the experimental campaign, six cellular structures were fabricated for each topology, resulting in a total of thirty specimens. Three specimens from each configuration were tested individually under uniaxial compression to characterize the intrinsic mechanical behavior of the individual cellular structures. The remaining three specimens were embedded in the mortar matrix and subsequently tested as internal reinforcement to evaluate the influence of the cellular topology on the mechanical performance of the resulting composite material. Consequently, all experimental conditions were evaluated using three independent replicates, which allowed the mechanical properties to be reported as average values and ensured the statistical reliability of the experimental results.

### 2.2. Fabrication of Mortar Composite Specimens

The mortar was prepared using ordinary Portland cement, natural fine siliceous aggregate, and potable water. A water-cement-sand ratio of 0.75:1:3 by mass was used, following standard laboratory procedures for hydraulic cement mortars in accordance with ASTM C305 [51]. The constituent materials were mechanically mixed until a homogeneous and workable mortar was obtained.

**Figure 3:**
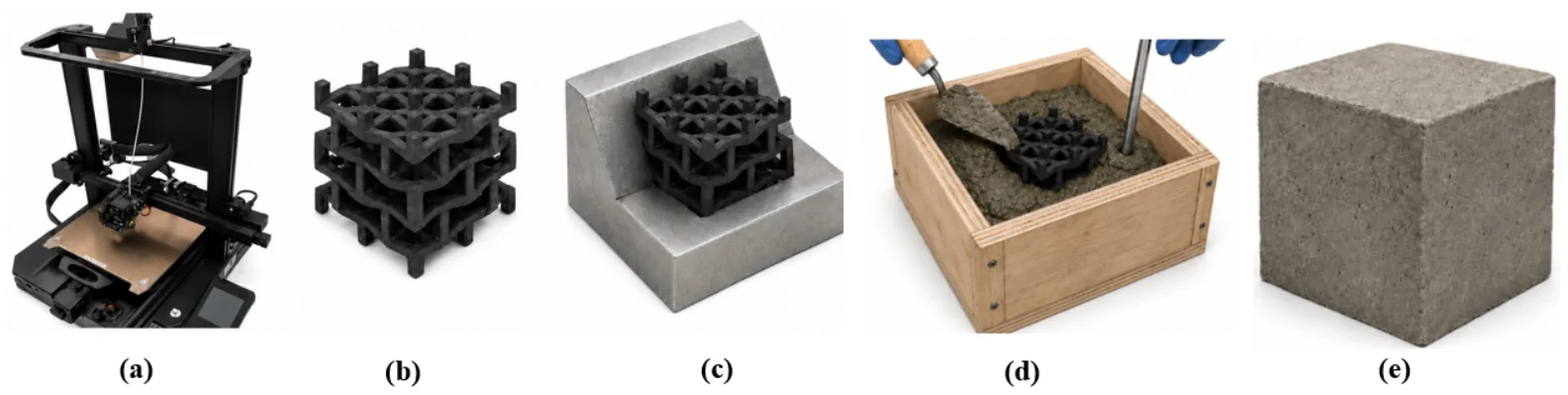
(**a**) fused-deposition-modeling system used to manufacture the PLA lattices; (**b**) representative printed lattice; (**c**) positioning and alignment of the lattice within the mold; (**d**) mortar placement and compaction around the lattice; and (**e**) representative completed mortar-lattice specimen after curing.

**Figure 4:**
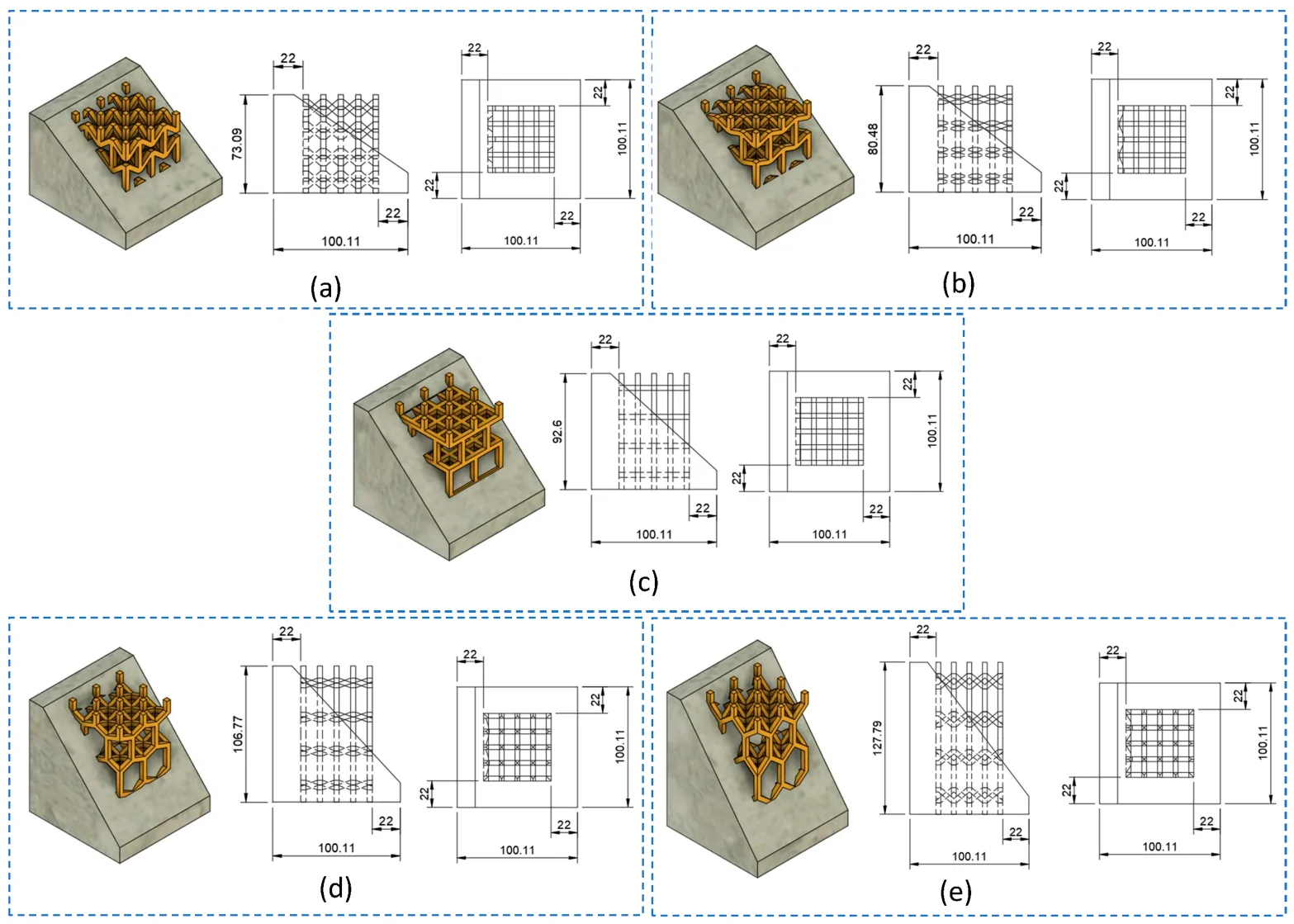
Geometrical configuration of the mortar specimens reinforced with embedded PLA cellular architectures: (**a**) re-entrant $-50^{\circ }$, (**b**) re-entrant $-70^{\circ }$, (**c**) cubic $90^{\circ }$, (**d**) honeycomb $+70^{\circ }$, and (**e**) honeycomb $+50^{\circ }$. Isometric and orthographic views illustrating the embedded lattice arrangement, specimen dimensions, and mortar cover. All dimensions are expressed in millimeters (mm).

Immediately after mixing, the 3D-printed cellular reinforcements were placed inside custom-designed molds with constant planar dimensions of 100 × 100 mm, adjusting the mold height according to the height of each cellular reinforcement. Each reinforcement was carefully centered within the mold to provide uniform mortar coverage of 22 mm on each lateral face, as illustrated in Figure 3. Maintaining constant lateral mortar coverage provided comparable matrix coverage and geometric boundary conditions for all specimens, thereby reducing variability unrelated to cellular topology. Since the total height of the cellular reinforcements varied with the strut angle, no mortar coating was applied to either the top or bottom surfaces, as illustrated in Figure 4. This configuration allowed the applied compressive load to be transferred simultaneously through the mortar matrix and the cellular reinforcement, thereby replicating the expected load-sharing mechanism of the composite under compression, while maintaining identical loading conditions for all the topologies investigated.

All composite specimens were cast in a single batch using the same mortar mix to ensure material homogeneity throughout the experimental campaign. The fresh mortar was poured around the cellular reinforcement and compacted using a vibrating table to ensure complete filling of the cellular cavities and minimize the presence of trapped air. After casting, the specimens remained in their molds for 48 h under laboratory conditions before being demolded. Subsequently, all specimens were immersed in a water curing tank for 24 days under controlled laboratory conditions. After 26 days, the specimens were removed from the curing tank and allowed to dry at laboratory ambient temperature. Finally, all composite specimens were subjected to uniaxial compression tests at 28 days.

In addition to the reinforced specimens, three unreinforced mortar specimens were fabricated as reference material. These control specimens had the same dimensions (100 × 100 mm) as the reinforced composites, while their height was set at 92.6 mm, corresponding to the height of the cubic cellular configuration ($\theta =90^{\circ }$). This geometry was selected because it represents an intermediate height among the five topologies investigated, providing a single representative reference sample and avoiding the introduction of an additional experimental variable associated with sample height. Consequently, the comparison between the reinforced and unreinforced samples primarily reflects the effect of the integrated cellular architecture, rather than differences in sample geometry.

Prior to the mechanical tests, the actual dimensions of all samples were determined using a digital caliper, and their mass was measured using a precision digital scale. These measurements were used to calculate the bulk density of each sample, which allowed for the accurate characterization of their geometric and physical properties prior to testing. The measured dimensions, mass, and calculated density of the unreinforced mortar samples, the standalone cellular structures, and the composite samples consisting of mortar and a cellular structure are summarized in Table 1.

### 2.3. Mechanical Characterization Under Compression

**Figure 5:**
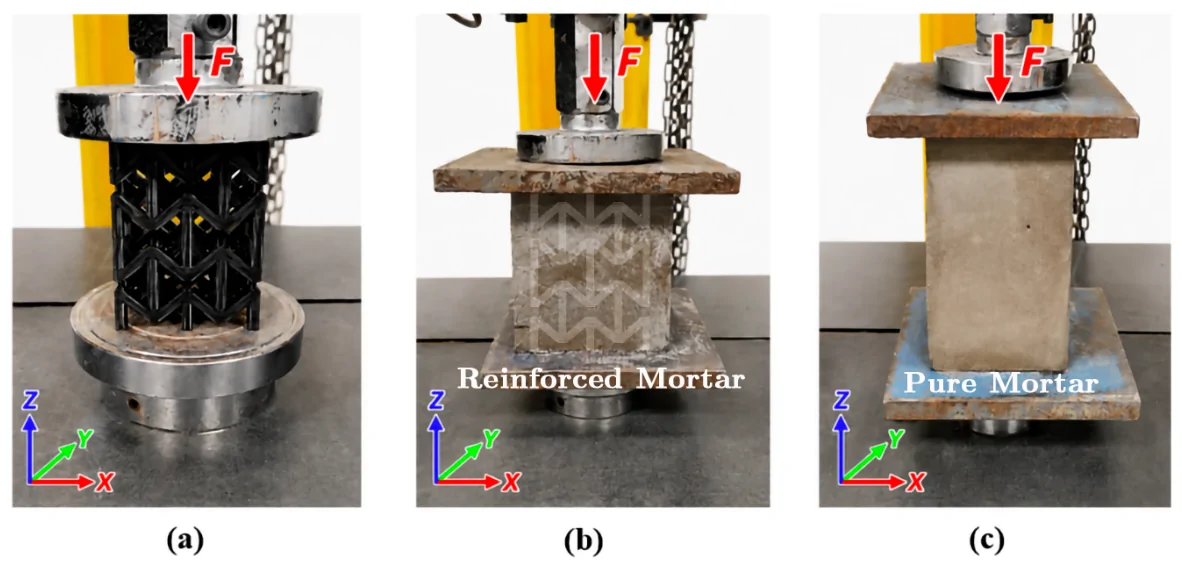
Quasi-static axial compression-test configurations: (**a**) standalone PLA lattice; (**b**) mortar specimen containing an embedded PLA lattice; and (**c**) unreinforced mortar control. The loading direction and platen arrangement are indicated.

The compressive behavior of the standalone cellular structures, the pure mortar specimens, and the composite specimens consisting of mortar and a cellular structure was evaluated using a Laryee UE3410 universal testing machine (Laryee Technology Co., Ltd., Beijing, China) equipped with a 100 kN load cell. The three test configurations investigated in this study are illustrated in Figure 5.

All tests were conducted under displacement-controlled loading at a constant crosshead speed of 5 mm/min. Although the height of the specimens varied among the cellular topologies investigated, the same crosshead speed was used for all tests to ensure identical experimental conditions throughout the testing program. Since the reinforced specimens incorporated cellular structures with different geometries and heights, the objective of the experimental campaign was to comparatively evaluate the influence of the integrated cellular architecture on the composite material’s compressive behavior, rather than to determine the standardized compressive strength of the mortar. Therefore, all specimens were tested under identical loading conditions, which made it possible to isolate and directly compare the effect of the cellular topology.

During each test, the testing machine continuously recorded the applied compressive load and the displacement of the load head. Subsequently, the engineering stress and strain were calculated from the measured force, the initial cross-sectional area under load, the recorded displacement, and the initial height of the specimen. The resulting stress–strain curves were used to determine the apparent compressive modulus, apparent yield stress, energy absorption density (*EA*), and specific energy absorption (*SEA*) of each specimen.

Young’s modulus was determined from the initial linear portion of the stress-strain curve. The yield stress was identified using the conventional 0.2% offset method for specimens exhibiting a well-defined post-elastic response. In this procedure, a line parallel to the initial linear elastic region was shifted by a strain offset of 0.2%, and the intersection between this offset line and the experimental stress-strain curve was taken as the yield stress. This criterion was applied to the standalone PLA cellular structures and to the mortar-cellular composite specimens whenever a distinct yield behavior was observed. Conversely, for specimens that exhibit brittle failure without a discernible yield point—such as unreinforced mortar specimens—the maximum compressive stress was adopted as the apparent yield stress for comparative purposes.

Energy absorption density (*EA*) was determined as the area under the experimental engineering stress–strain curve between the onset of yielding and the terminal strain used for the analysis. It therefore represents the mechanical energy dissipated per unit initial specimen volume during post-yield deformation and progressive structural damage:

(1) $$EA=\int_{\varepsilon_{y}}^{\varepsilon_{u}}\sigma \left(\varepsilon \right)\;d\varepsilon$$

where σ(ε) is the engineering compressive stress, $\varepsilon_{y}$ is the strain corresponding to the apparent yield point, and $\varepsilon_{u}$ is the terminal strain used as the upper integration limit. For specimens exhibiting brittle behavior, $\varepsilon_{u}$ corresponded to the strain at maximum stress; for specimens exhibiting progressive post-yield deformation, $\varepsilon_{u}$ corresponded to the last recorded point prior to complete structural collapse. Because strain is dimensionless, the integral has units of energy per unit volume and is reported as MJ/m^3^; numerically, 1 MPa is equivalent to 1 MJ/m^3^.

Because energy absorption density does not account for differences in specimen mass, it was additionally normalized by the specimen bulk density to obtain the specific energy absorption (*SEA*):

(2) $$SEA=\frac{EA}{\rho }$$

where ρ is the specimen bulk density. With *EA* expressed in MJ/m^3^ and ρ in g/cm^3^, *SEA* is obtained in J/g. Each test configuration was evaluated using three independent specimens, and the reported mechanical properties correspond to mean values with their respective standard deviations. In total, 33 compression tests were conducted, comprising five standalone cellular configurations, five mortar–cellular composite configurations, and one unreinforced mortar control.

### 2.4. Statistical Analysis

All mechanical properties are reported as mean ± standard deviation from three independent specimens (n=3) for each configuration. The effect of cellular topology on each mechanical response of the reinforced composites was evaluated using one-way analysis of variance (ANOVA) with a significance level of α=0.05. When the omnibus ANOVA indicated a statistically significant topology effect, Tukey’s honestly significant difference (HSD) procedure was used for post hoc pairwise comparisons. For direct comparisons involving the unreinforced mortar reference, the mortar group was included as an additional comparison group. Statistical significance is reported explicitly using *p*-values; elsewhere, descriptive terms such as “higher”, “lower”, or “substantial” are used without implying inferential significance.

## 3. Results and Discussion

The experimental results are presented step by step to facilitate the interpretation of the role played by the integrated 3D-printed cellular architectures in the compressive behavior of the developed composite specimens. First, the compressive response of unreinforced mortar is presented as a reference for the cementitious matrix. Next, the mechanical behavior of the standalone cellular structures is analyzed to determine the influence of different lattice topologies independently of the surrounding mortar. Subsequently, the response of the composite mortar-cellular specimens is analyzed to evaluate the interaction between the printed reinforcement and the cementitious matrix under compressive loading. Finally, the influence of cellular topology on overall mechanical performance is evaluated comparatively using the measured apparent compressive modulus, apparent yield stress, energy absorption density (*EA*), and specific energy absorption (*SEA*), providing a comprehensive assessment of the effectiveness of each reinforcement architecture.

### 3.1. Compressive Response of the Reference Mortar

**Figure 6:**
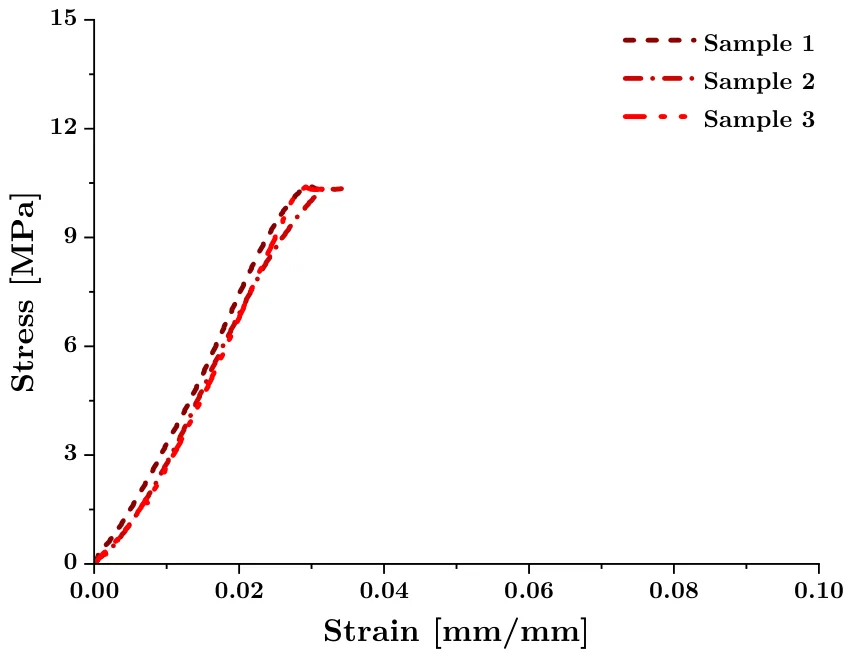
Experimental stress–strain response of the mortar control samples under compressive loading.

Figure 6 shows the stress-strain curves obtained from the three unreinforced mortar specimens subjected to uniaxial compression. The experimental results showed excellent agreement throughout the entire loading process, with stress-strain curves that were virtually superimposed and minimal variation in the measured mechanical properties. Young’s modulus and the ultimate stress reached 389.66 ± 3.48 MPa and 10.41 ± 0.01 MPa, respectively, corresponding to coefficients of variation of less than 1% for both parameters. This low experimental variability demonstrates the robustness of the sample preparation procedure, the curing conditions, and the testing protocol, thereby providing a highly reliable mechanical reference for the subsequent evaluation of the reinforced samples.

Rather than providing an isolated characterization of the material, the mechanical response of plain mortar establishes the experimental basis needed to quantify the contribution of the integrated cellular architectures. Because cementitious materials exhibit variability associated with their heterogeneous microstructure, obtaining a highly reproducible control mechanical response is essential to ensure that any subsequent changes in mechanical behavior can be attributed to the reinforcement topology and not to experimental variation in the mortar matrix. Therefore, the excellent repeatability observed in the reference samples enhances the reliability of the comparisons made throughout this study. As expected, the plain mortar exhibited linear elastic behavior, followed by a sudden loss of load-bearing capacity after reaching maximum stress, indicating zero deformation beyond the elastic limit. Although this failure mode is well documented in cementitious materials, its characterization in this study is particularly relevant, as it establishes the deformation regime that the incorporated cellular reinforcements are intended to improve. Therefore, the objective of incorporating 3D-printed cellular structures is not only to alter the composite’s initial stiffness or load-bearing capacity but also to promote progressive deformation after yield and, consequently, to increase the mechanical energy dissipated before complete structural collapse.

Thus, Figure 6 establishes the control mechanical response against which the influence of the different cellular topologies is evaluated in the following sections. This reference allows not only for a quantitative comparison of stiffness and strength but also for an evaluation of each reinforcement architecture’s ability to improve the brittle response of plain mortar in a composite material with greater damage tolerance.

### 3.2. Mechanical Response of the Standalone Cellular Structures

**Figure 7:**
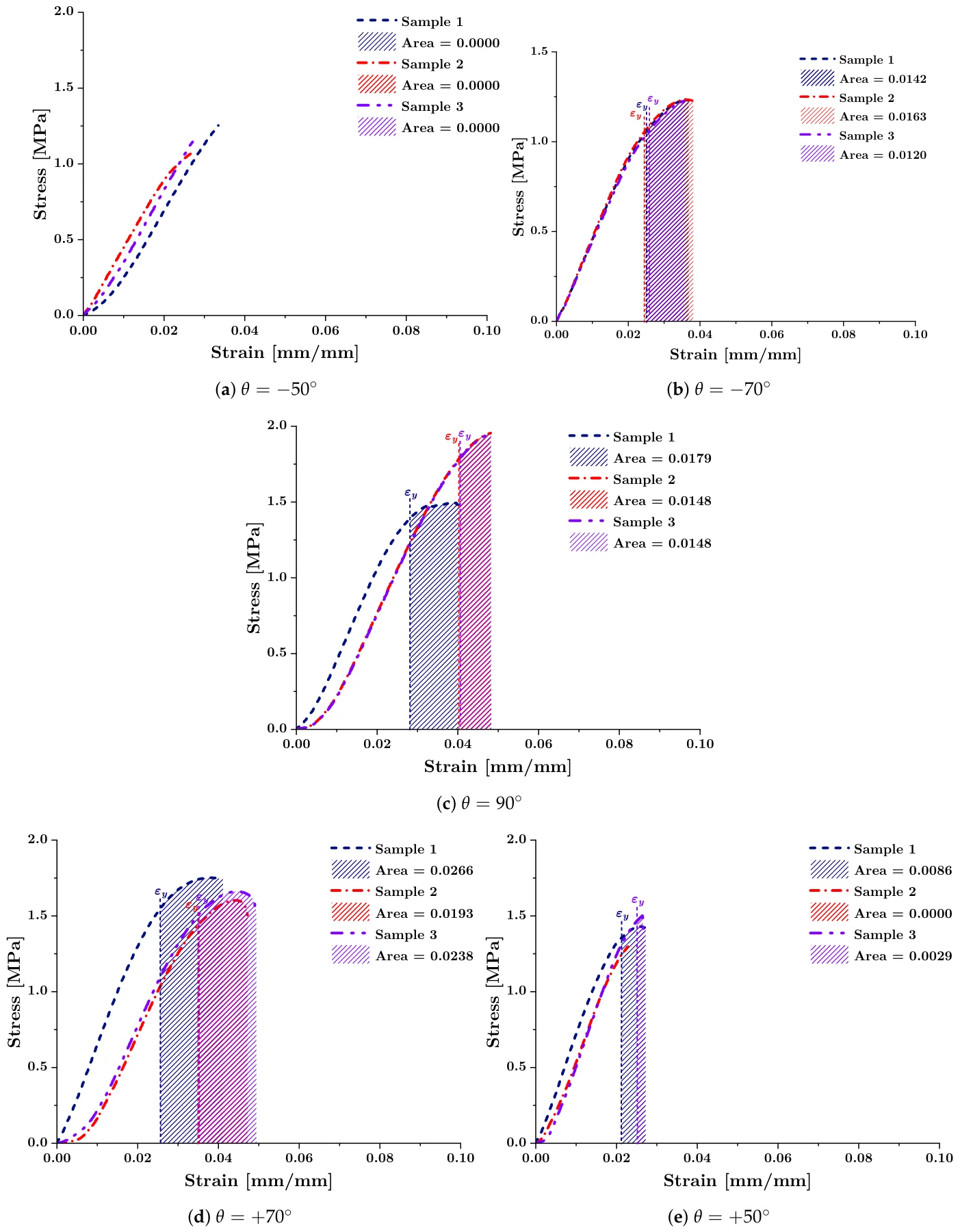
Compressive stress–strain behavior of the 3D-printed cellular structures with different strut angles. The investigated geometries comprise auxetic ($\theta =-70^{\circ }$ and $-50^{\circ }$), cubic ($\theta =90^{\circ }$), and honeycomb ($\theta =+70^{\circ }$ and $+50^{\circ }$) architectures. Three experimental replicates are shown for each configuration. $\varepsilon_{y}$ denotes the strain corresponding to the apparent yield point determined using the 0.2% offset criterion, whereas $\varepsilon_{u}$ denotes the terminal strain used as the upper limit for calculation of the post-yield energy absorption density. The shaded region between $\varepsilon_{y}$ and $\varepsilon_{u}$ represents the energy absorption density calculated from the stress–strain response. The quantities labeled “Area” within the original plot panels correspond to this *EA* value.

Figure 7 shows the stress–strain curves obtained from the 3D-printed cellular structures subjected to uniaxial compression. For each topology, the three experimental replicates showed excellent agreement throughout the loading history, demonstrating the high repeatability of both the manufacturing process and the compression tests. The low dispersion observed in the stress-strain responses confirms that the measured differences among the investigated architectures stem primarily from the cellular topology and not from manufacturing variability.

The overall mechanical response differed considerably among the five architectures studied. Young’s modulus increased from 45.17 MPa for the auxetic structure ($-50^{\circ }$) to 71.06 MPa for the honeycomb configuration ($+50^{\circ }$), while the apparent yield stress ranged from 1.06 MPa to 1.65 MPa. Despite these differences in stiffness and strength, all standalone structures exhibited relatively limited post-yield deformation and low energy absorption density. In particular, the auxetic ($-50^{\circ }$) and honeycomb ($+50^{\circ }$) specimens showed abrupt stress drops with little or no post-peak plateau. The remaining architectures exhibited a somewhat more progressive response; nevertheless, the highest mean *EA* among the standalone lattices was only 0.023 ± 0.004 MJ/m^3^.

It can be observed that this mechanical behavior appears to contradict the extensive literature reporting superior energy-absorption capabilities for auxetic and honeycomb cellular structures. Numerous experimental and numerical studies have demonstrated that these architectures are capable of dissipating significant amounts of mechanical energy through progressive collapse mechanisms involving strut bending, nodal rotation, and sequential buckling of the cells [52,53]. However, most of these studies also emphasize that the effectiveness of these deformation mechanisms depends largely on the manufacturing process and, in particular, on the integrity of the printed struts [29,54].

In this study, all cellular structures were fabricated using fused deposition modeling (FDM), with the specimens printed on the xy build platform. FDM fabrication is known to introduce direction-dependent mechanical properties because bonding between successive deposited layers is generally weaker than the continuous filament material [55,56,57]. Given the printing orientation adopted here, these interfaces may therefore contribute to the abrupt stress drops observed in some standalone lattices. The experimental stress–strain curves are consistent with premature local failure interrupting the progressive bending, rotation, and buckling mechanisms normally associated with cellular architectures. However, because the fracture surfaces were not characterized microscopically after testing, the specific contribution of interlayer delamination to the present response cannot be established directly.

Accordingly, the relatively low energy absorption density of the isolated lattices should not be interpreted as an intrinsic limitation of the investigated topologies. Rather, it reflects the combined effects of architecture and FDM-induced anisotropy under the unconstrained boundary conditions of the standalone tests.

More importantly, the standalone and embedded lattices experience different mechanical boundary conditions. The isolated structures permit comparatively unconstrained lateral deformation, whereas the surrounding mortar provides distributed mechanical support and participates in load transfer. Incorporation into mortar therefore changes the stress paths and deformation modes of the cellular network. The mechanical properties of an isolated FDM lattice should consequently not be directly extrapolated to the corresponding mortar–lattice composite.

### 3.3. Mechanical Performance of Mortar–Cellular Composite Specimens

**Figure 8:**
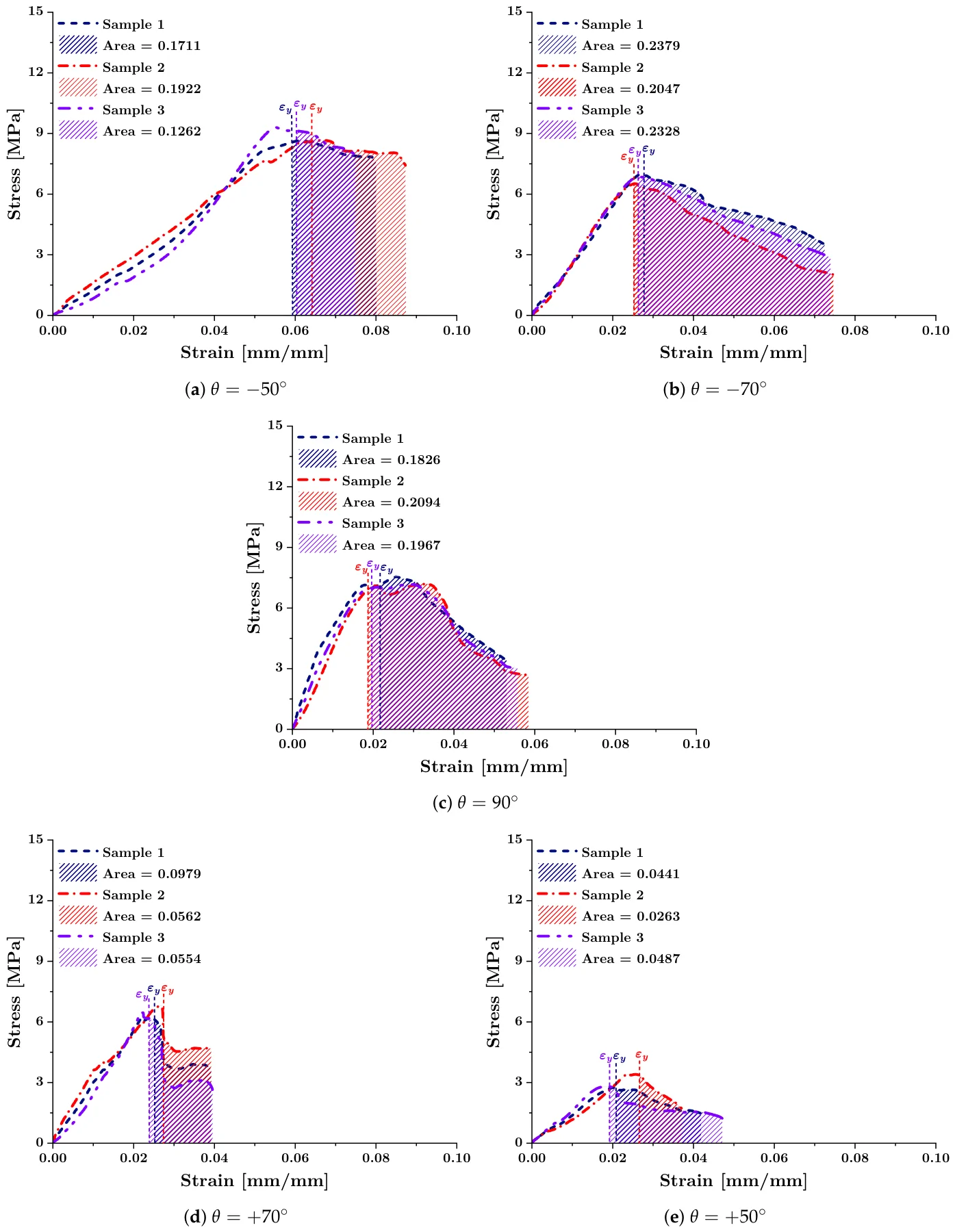
Compressive stress–strain response of mortar specimens reinforced with different 3D-printed cellular architectures: (**a**) auxetic $\theta =-50^{\circ }$, (**b**) auxetic $\theta =-70^{\circ }$, (**c**) cubic $\theta =90^{\circ }$, (**d**) honeycomb $\theta =+70^{\circ }$, and (**e**) honeycomb $\theta =+50^{\circ }$. $\varepsilon_{y}$ denotes the strain corresponding to the apparent yield point determined using the 0.2% offset criterion, whereas $\varepsilon_{u}$ denotes the terminal strain used as the upper integration limit for the post-yield energy absorption density. The shaded region between $\varepsilon_{y}$ and $\varepsilon_{u}$ represents the corresponding energy absorption density. The quantities labeled “Area” within the original plot panels correspond to this *EA* value.

Figure 8 shows the stress-strain response of the mortar specimens reinforced with the five 3D-printed cellular architectures investigated. Similar to the reference mortar and the independent lattice structures, the three experimental replicates corresponding to each reinforcement topology showed good agreement among the three tested replicates. The low experimental dispersion confirms the high repeatability of the sample fabrication procedure and indicates that the observed differences are primarily associated with the geometry of the integrated cellular reinforcement, rather than with variability in the mortar matrix.

Compared with the standalone cellular structures, the reinforced specimens exhibited a more gradual post-peak response and retained load-bearing capacity over a larger strain interval. At the macroscopic level, this behavior is consistent with a passive-confinement effect imposed by the surrounding mortar. In this context, passive confinement refers to mechanical restraint provided by the matrix without externally applied prestress: the mortar can restrict lateral motion of inclined members, provide distributed bearing around struts and nodes, and establish additional load-sharing paths between the cellular reinforcement and the cementitious phase. These effects would be expected to reduce highly localized deformation and allow compressive loads to be redistributed over a larger portion of the embedded network, thereby extending bending, nodal rotation, and progressive cellular collapse over a larger strain interval.

Nevertheless, the present experiments provide macroscopic mechanical evidence only. No post-test optical or scanning electron microscopy, full-field strain measurements, or internal crack mapping was performed. Consequently, the experiments do not directly establish whether FDM interlayer cracking was delayed, whether PLA–mortar debonding or delamination occurred, or how cracking developed within the surrounding mortar. Passive confinement is therefore proposed as a mechanistic interpretation consistent with the measured stress–strain response, rather than as a directly observed microscale failure mechanism. The reinforced response should accordingly be interpreted as arising from matrix–lattice interaction rather than as a simple superposition of the properties of the two constituents.

The following section provides a quantitative comparison of the influence of each cellular topology on apparent stiffness, apparent yield stress, energy absorption density, and specific energy absorption.

### 3.4. Effect of Cellular Topology on the Mechanical Performance

**Figure 9:**
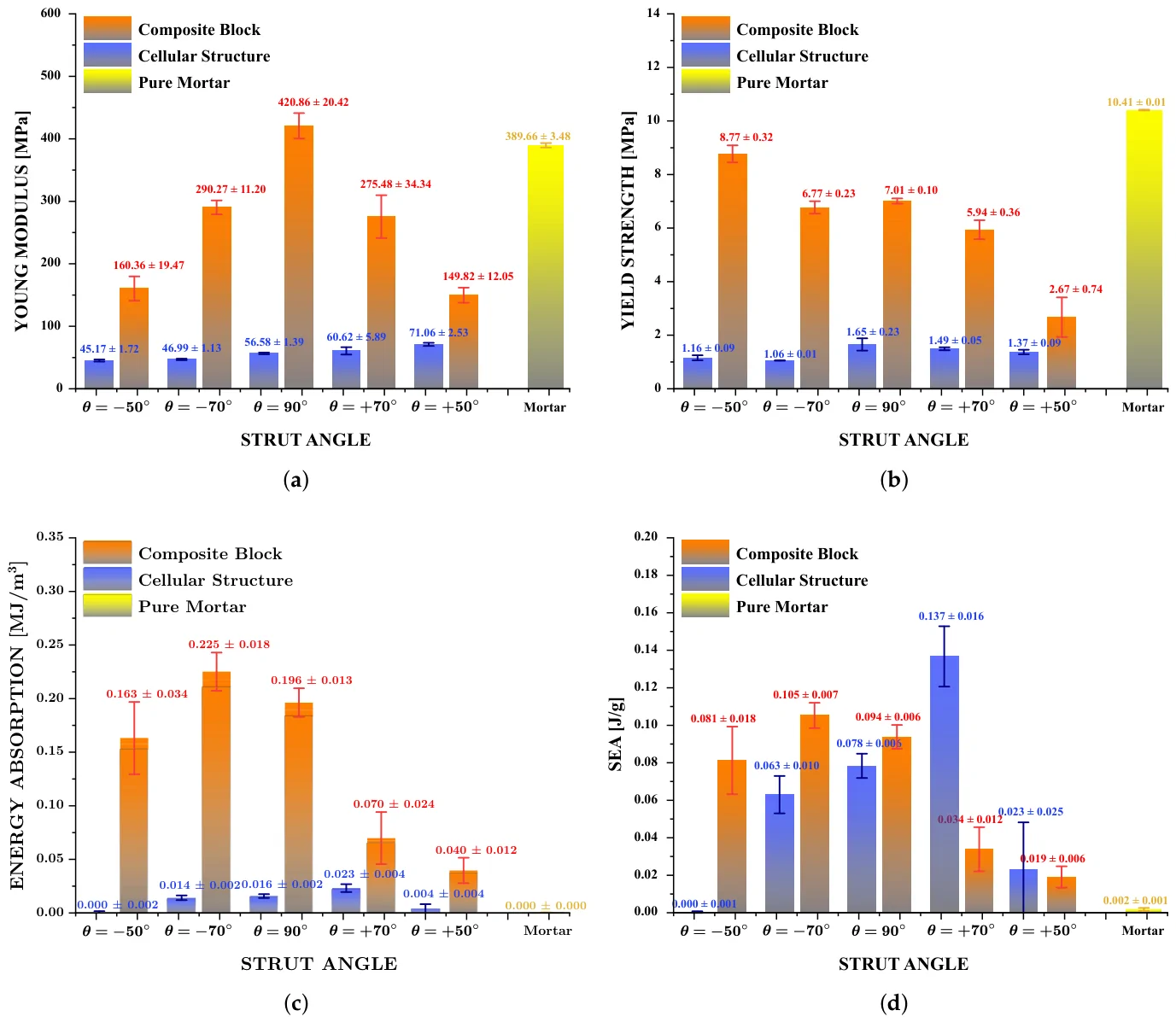
Comparison of the mechanical properties of the standalone 3D-printed cellular structures, mortar–cellular structure composites, and unreinforced mortar: (**a**) apparent compressive modulus, (**b**) apparent yield stress, (**c**) energy absorption density (*EA*), and (**d**) specific energy absorption (*SEA*). Values are reported as mean ± standard deviation (n=3).

Figure 9 compares the mechanical behavior of standalone cellular structures, composite specimens consisting of mortar and cellular structures, and unreinforced mortar in terms of apparent compressive modulus, apparent yield stress, energy absorption density (*EA*), and specific energy absorption (*SEA*). Unlike the stress-strain curves analyzed in the previous sections, these results allow for a direct quantitative assessment of the influence of cellular topology on the overall mechanical behavior of the composite system. The comparison demonstrates that the mechanical response is determined not only by the geometry of the printed cellular structure but also by its interaction with the surrounding mortar matrix.

In addition, a statistical analysis was performed using one-way analysis of variance (ANOVA) to evaluate the effect of the cellular configuration on the measured mechanical properties, as shown in Table 2. Separate analyses were conducted for the mortar-reinforced samples and the standalone PLA cellular structures. The configurations investigated were considered independent groups, with three samples analyzed for each configuration (n=3). When statistically significant differences were identified, Tukey’s post-hoc Honest Significant Difference (HSD) test was used for pairwise comparisons. Statistical significance was assessed at a significance level of p<0.05. In addition to *p*-values, effect size was quantified using eta-squared ($\eta^{2}$).

The influence of cellular topology on the apparent compressive modulus is shown in Figure 9a. One-way ANOVA confirmed a significant effect of cellular configuration among the reinforced composites (F(4,10)=81.99, p<0.0001). The cubic configuration ($90^{\circ }$) exhibited the highest mean apparent compressive modulus (420.86 ± 20.42 MPa), which was 8.0% higher than the mean value of plain mortar (389.66 ± 3.48 MPa). However, in the comparison including the mortar control, this pairwise difference was not statistically significant (Tukey HSD, p=0.411); the 8.0% difference is therefore interpreted descriptively rather than as a statistically demonstrated stiffness increase. The comparatively high modulus of the cubic architecture is consistent with its more direct compressive load paths and greater contribution of axial deformation. In contrast, the auxetic configuration ($-70^{\circ }$) maintained a mean modulus of 290.27 MPa, whereas the honeycomb configuration ($+50^{\circ }$) exhibited the lowest mean stiffness (149.82 MPa). These results show that strut orientation markedly changes the efficiency of initial load transfer within the composite system.

Figure 9b shows that apparent yield stress followed a different trend from apparent compressive modulus. The effect of cellular configuration among the reinforced composites was statistically significant (F(4,10)=106.30, p<0.0001). Although none of the reinforced configurations exceeded the mean apparent yield stress of plain mortar (10.41 MPa), the auxetic configuration ($-50^{\circ }$) achieved the highest mean value among the reinforced composites (8.77 MPa), corresponding to approximately 84% of the mortar reference. The honeycomb configuration ($+50^{\circ }$) exhibited the lowest mean apparent yield stress (2.67 MPa). These results indicate that the configuration maximizing initial stiffness is not necessarily the configuration providing the highest load-bearing capacity before the onset of post-yield deformation.

The differences among the investigated configurations may be interpreted in terms of the deformation modes promoted by strut orientation. Re-entrant architectures may provide greater geometric freedom for strut rotation and bending, potentially enabling progressive deformation over a larger strain interval. By contrast, the cubic architecture provides more direct load paths in the compression direction, increasing the contribution of axial deformation and consequently contributing to its higher initial stiffness. This interpretation is consistent with the observation that the cubic configuration exhibited the highest mean apparent compressive modulus without simultaneously maximizing energy absorption. Energy dissipation depends on the extent and stability of the post-yield deformation response rather than exclusively on initial stiffness; accordingly, the $-70^{\circ }$ auxetic configuration exhibited the highest energy absorption density. Thus, the strut angle appears to influence a trade-off between efficient initial load transfer and post-yield deformation capacity.

The differences between the architectures become more evident when evaluating the response following the load peak. As shown in Figure 9c, cellular configuration significantly affected energy absorption density among the reinforced composites (F(4,10)=41.08, p<0.0001). The unreinforced mortar exhibited essentially zero post-yield energy absorption under the adopted definition, whereas all reinforced configurations dissipated measurable mechanical energy before collapse. The $-70^{\circ }$ auxetic configuration exhibited the highest mean energy absorption density (0.225 ± 0.018 MJ/m^3^), followed by the cubic configuration (0.196 ± 0.013 MJ/m^3^) and the $-50^{\circ }$ auxetic configuration (0.163 ± 0.034 MJ/m^3^). The honeycomb architectures exhibited lower mean values, particularly the $+50^{\circ }$ configuration.

A comparison of specific energy absorption (Figure 9d) further demonstrates the influence of cellular configuration on the energy dissipation efficiency. The effect of cellular configuration among the reinforced composites was statistically significant (F(4,10)=87.00, p<0.0001). For the standalone lattices, the honeycomb architecture ($+70^{\circ }$) exhibited the highest mean specific energy absorption (0.137 ± 0.015 J/g), whereas, after incorporation into mortar, the auxetic configuration ($-70^{\circ }$) became the most efficient reinforced configuration (0.105 ± 0.007 J/g). This reversal in the ranking of the investigated configurations indicates that the mechanical response of an isolated FDM-fabricated lattice cannot be directly extrapolated to its corresponding mortar-integrated system. The surrounding mortar modifies the interaction between the cellular reinforcement and the composite system, thereby altering the overall load-transfer and energy-dissipation response.

No single cellular architecture maximized apparent stiffness, apparent yield stress, and energy absorption simultaneously because these responses are governed by different deformation requirements. Initial stiffness benefits from efficient and relatively direct load transfer with limited geometric deformation, whereas energy absorption requires sustained post-yield deformation and progressive structural rearrangement. The onset of yielding is additionally controlled by local stress concentrations and the initiation of geometric instability. Accordingly, topology selection should be application-specific. Under the quasi-static loading conditions investigated here, the cubic architecture is preferred when initial stiffness is prioritized, the $-50^{\circ }$ auxetic topology provides the highest apparent yield stress among the reinforced composites, and the $-70^{\circ }$ auxetic topology provides the highest energy absorption density and specific energy absorption. These results therefore define a property-dependent design space rather than a single universally optimal architecture.

The ranking of the isolated networks also changed substantially after incorporation into the mortar matrix. For example, the $+50^{\circ }$ honeycomb lattice exhibited the highest apparent compressive modulus among the standalone structures (71.06 MPa) but produced the least stiff composite (149.82 MPa). Conversely, the $-70^{\circ }$ auxetic lattice exhibited one of the lowest standalone stiffness values (46.99 MPa) but became one of the higher-stiffness reinforced configurations. This ranking reversal provides direct macroscopic evidence that topology must be evaluated within the composite system for which it is intended.

The change in topology ranking between the isolated lattices and the corresponding composites demonstrates that the surrounding mortar modifies the effective mechanical boundary conditions of the cellular reinforcement. Distributed support and load sharing by the matrix are consistent with the more gradual post-yield responses observed experimentally. However, because the PLA–mortar interface and internal fracture processes were not directly characterized, the respective contributions of matrix cracking, interface debonding, FDM-layer separation, and geometric lattice instability cannot be distinguished from the present measurements. Passive confinement should therefore be regarded as a mechanically consistent interpretation rather than a microscopically proven mechanism. The proposed cellular reinforcement differs from conventional approaches—such as steel, polypropylene, and basalt fibers, which primarily improve crack control by forming distributed bridges—and from textile reinforcements or polymer meshes, which provide continuous directional reinforcement. In contrast, the present approach introduces a three-dimensional reinforcement with a defined structure, whose response can be modified through its cellular configuration.

From an application perspective, the proposed mortar-lattice reinforcement system should currently be considered a proof of concept for the development of cementitious components with controlled deformation and enhanced energy dissipation under quasi-static compressive loading. Although these architectures may have potential for impact-related applications, such performance cannot be established from the present results and must be evaluated through dedicated dynamic and high-strain-rate tests.

PLA was selected due to its excellent printability and dimensional accuracy using conventional fused deposition modeling, which enables the reliable fabrication of complex cellular architectures. Furthermore, according to [31], PLA cellular reinforcements exhibited the highest stiffness and energy absorption capacity among the polymeric materials evaluated for mortar-reinforced cellular composites. However, the long-term durability of PLA in cementitious environments—including its behavior under alkaline exposure, humidity, sustained loading, and thermal variations—was not evaluated in this study and should be addressed in future research. While the incorporation of cellular reinforcement into the cementitious matrix can reduce its direct exposure to external environmental conditions and temperature fluctuations, it does not completely eliminate the potential for degradation associated with the alkaline environment within the pores or with long-term polymer aging. Therefore, the present results should be interpreted primarily as evidence of the short-term mechanical behavior of PLA cellular reinforcements with controlled topology, while specific durability studies will be required before considering long-term structural applications.

Thus, this study was limited to quasi-static compression, which was selected as a proof-of-concept test method to evaluate the influence of the cellular configuration on the mechanical response and energy dissipation of the mortar-truss composite. However, compression alone does not allow for determining the overall effectiveness of the proposed system’s reinforcement, especially since the mortar is strongly affected by tensile and flexural cracking. Therefore, future studies should evaluate the behavior under tension, bending, fracture, and impact.

## 4. Conclusions

This study experimentally investigated the influence of 3D-printed PLA cellular architectures on the quasi-static compressive behavior of mortar composites. Five lattice topologies—including auxetic, cubic, and honeycomb configurations—were evaluated to determine how internal architecture influences apparent stiffness, apparent yield stress, energy absorption density, and specific energy absorption. Based on the experimental results, the following conclusions can be drawn:

- Cellular topology exerted a statistically significant overall effect on the quasi-static mechanical response of the reinforced mortar composites, including apparent compressive modulus, apparent yield stress, energy absorption density, and specific energy absorption (all p<0.0001). The results confirm that internal architecture can be used to tailor mechanical response while maintaining an unchanged mortar formulation.
- The mechanical response of the standalone FDM-printed lattices differed substantially from that of the corresponding mortar–lattice composites. The isolated structures generally exhibited abrupt post-peak load losses, whereas the embedded lattices sustained load over larger post-yield strain intervals. The behavior of an isolated lattice therefore cannot be directly extrapolated to that of the corresponding composite.
- The more gradual post-yield response of the embedded structures is consistent with a passive-confinement effect associated with matrix restraint and load sharing. However, the present study did not include post-test microscopy, interface characterization, or full-field/internal damage measurements; therefore, interlayer cracking, PLA–mortar debonding or delamination, and internal crack trajectories were not directly resolved. The proposed confinement mechanism should consequently be regarded as a mechanically consistent interpretation requiring direct experimental validation.
- No single topology simultaneously maximized all mechanical responses. The cubic configuration exhibited the highest mean apparent compressive modulus, although its approximately 8% increase relative to plain mortar was not statistically significant (Tukey HSD, p=0.411); the $-50^{\circ }$ auxetic configuration exhibited the highest apparent yield stress among the reinforced composites; and the $-70^{\circ }$ auxetic configuration exhibited the highest energy absorption density and specific energy absorption. Topology selection should therefore depend on whether stiffness, load-bearing capacity, or quasi-static energy dissipation is the principal design criterion.

These results demonstrate that the quasi-static compressive response of cementitious composites can be tailored through internal cellular architecture without modifying the mortar formulation. Nevertheless, the conclusions are restricted to the geometries, relative densities, materials, manufacturing conditions, and quasi-static loading rate investigated here. Because all experiments were conducted at a crosshead speed of 5 mm/min, the present results should not be extrapolated directly to impact or other high-strain-rate conditions. Future work should combine post-test optical and scanning electron microscopy of the PLA–mortar interface, full-field strain measurements, internal damage characterization, numerical modeling, and dynamic testing to distinguish matrix cracking, interface debonding, interlayer failure, and lattice deformation mechanisms and to determine whether the observed quasi-static energy-dissipation advantages persist under high-rate loading.

## Figures and Tables

**Table 1:** Geometrical dimensions, mass, and bulk density of the mortar specimens, cellular structures, and mortar–structure composite specimens evaluated in this study.

Configuration	Sample	Length	Width	Height	Weight	Density
		[mm]	[mm]	[mm]	[g]	[g/cm^3^]
Pure Mortar	1	99.86	99.16	92.10	1973.25	2.16
Pure Mortar	2	99.71	99.24	92.86	1970.17	2.14
Pure Mortar	3	99.76	99.27	92.14	1965.17	2.15
Structure $\theta =-50^{\circ }$	1	55.14	55.19	73.85	69.15	0.31
Structure $\theta =-50^{\circ }$	2	55.13	55.20	73.84	65.03	0.29
Structure $\theta =-50^{\circ }$	3	55.10	55.13	73.80	64.09	0.29
Structure $\theta =-70^{\circ }$	1	55.19	55.20	80.85	58.98	0.24
Structure $\theta =-70^{\circ }$	2	55.19	55.28	80.80	54.13	0.22
Structure $\theta =-70^{\circ }$	3	55.19	55.29	80.90	53.14	0.22
Structure $\theta =90^{\circ }$	1	55.19	55.21	92.83	58.91	0.21
Structure $\theta =90^{\circ }$	2	55.10	55.23	92.86	55.26	0.20
Structure $\theta =90^{\circ }$	3	55.23	55.18	92.82	57.07	0.20
Structure $\theta =+70^{\circ }$	1	55.21	55.15	107.74	58.97	0.18
Structure $\theta =+70^{\circ }$	2	55.19	55.21	107.83	53.56	0.16
Structure $\theta =+70^{\circ }$	3	55.17	55.18	107.73	54.32	0.17
Structure $\theta =+50^{\circ }$	1	55.27	55.26	128.83	67.39	0.17
Structure $\theta =+50^{\circ }$	2	55.22	55.28	128.87	61.02	0.16
Structure $\theta =+50^{\circ }$	3	55.27	55.15	128.76	62.01	0.16
Mortar + Structure $\theta =-50^{\circ }$	1	99.78	101.85	75.70	1490.15	1.94
Mortar + Structure $\theta =-50^{\circ }$	2	99.87	100.87	75.14	1537.19	2.03
Mortar + Structure $\theta =-50^{\circ }$	3	98.73	99.82	74.27	1520.19	2.08
Mortar + Structure $\theta =-70^{\circ }$	1	99.88	99.89	84.88	1802.28	2.13
Mortar + Structure $\theta =-70^{\circ }$	2	98.86	99.15	82.28	1680.13	2.08
Mortar + Structure $\theta =-70^{\circ }$	3	97.86	98.14	83.10	1756.28	2.20
Mortar + Structure $\theta =90^{\circ }$	1	99.85	99.82	94.82	1978.19	2.09
Mortar + Structure $\theta =90^{\circ }$	2	99.80	100.73	94.73	1995.14	2.10
Mortar + Structure $\theta =90^{\circ }$	3	99.70	100.83	94.87	1991.17	2.09
Mortar + Structure $\theta =+70^{\circ }$	1	99.82	99.71	109.80	2255.26	2.06
Mortar + Structure $\theta =+70^{\circ }$	2	99.72	99.79	109.75	2247.20	2.06
Mortar + Structure $\theta =+70^{\circ }$	3	98.29	99.82	108.77	2207.29	2.07
Mortar + Structure $\theta =+50^{\circ }$	1	100.15	100.82	132.27	2741.10	2.05
Mortar + Structure $\theta =+50^{\circ }$	2	99.90	99.86	131.25	2747.15	2.10
Mortar + Structure $\theta =+50^{\circ }$	3	99.85	99.71	131.78	2764.29	2.11

**Table 2:** Results of one-way ANOVA for the investigated mechanical properties.

Specimen Group	Mechanical Property	F(4,10)	*p*-Value	η ^2^
Mortar + Structures	Young modulus	81.99	<0.0001	0.970
	Yield stress	106.3	<0.0001	0.977
	Energy absorption	41.08	<0.0001	0.943
	SEA	87.00	<0.0001	0.972
Structures	Young’s modulus	35.5	<0.0001	0.934
	Yield stress	14.66	0.00035	0.854
	Energy absorption	33.07	<0.0001	0.930
	SEA	43.27	<0.0001	0.945

## Data Availability

The original contributions presented in this study are included in the article. Further inquiries can be directed to the corresponding authors.
